# Thermal Lamination of Electrospun Nanofiber Membrane with Woven Fabric and Yarn Embedding Effect

**DOI:** 10.3390/membranes15030095

**Published:** 2025-03-20

**Authors:** Ziyuan Gao, Le Xu, Hongxia Wang, Xin Wei, Kaikai Chen, Wenyu Wang, Suzhen Zhang, Tong Lin

**Affiliations:** 1 School of Textile Science and Engineering, Tiangong University, Tianjin 300387, China; gzy15873494531@163.com (Z.G.); xlxule@163.com (L.X.); xin.wei@tiangong.edu.cn (X.W.); chenkaikai@tiangong.edu.cn (K.C.); wwy-322@126.com (W.W.); 2 The Key Laboratory of Hollow Fiber Membrane Materials and Membrane Processes, Ministry of Education, Tiangong University, Tianjin 300387, China; 3 Research Institute of Cangzhou, Tiangong University, Cangzhou 061014, China; 4 School of Chemical Engineering and Technology, Tiangong University, Tianjin 300387, China; zhangsztg@163.com

**Keywords:** nanofiber membrane, fabric, lamination, yarn embedding, air permeability, oil aerosol filtration

## Abstract

This study investigated the effectiveness of two lamination methods for integrating electrospun nanofiber membranes with woven nylon fabric for personal protective applications. The first method used a thermoplastic urethane (TPU) nonwoven adhesive, while the second method incorporated both the adhesive and a yarn, with the yarn embedding by sewing. Lamination with the TPU nonwoven adhesive slightly improved the adhesion between the nanofiber membrane and the nylon fabric. However, it decreased the air permeability, with the degree of the decrease depending on the areal density of the TPU adhesive. As the areal density of the TPU increased from 10 g/m^2^ to 30 g/m^2^, the air permeability decreased from 107.6 mm/s to 43.4 mm/s. The lamination resulted in a slight increase in the filtration efficiency for oil aerosol particles (0.3 µm, PM0.3, at a flow rate of 32 L/min) to 96.4%, with a pressure drop of 83 Pa. Embedding non-fusible yarns in the laminate increased the nanofiber/fabric adhesion and permeability. Still, the filtration efficiency and pressure drop were reduced to 74.4% and 38 Pa, respectively, due to numerous pinholes formed in the nanofiber layer during the sewing process. Conversely, incorporating fusible TPU yarns not only improved the interlayer adhesion by 175% compared to using TPU fabric adhesive alone but also increased the air permeability to 136.1 mm/s. However, the filtration performance (87.7%, 72 Pa) was slightly lower than that of the unlaminated nanofiber/fabric pack because the TPU yarns sealed the pinholes during lamination. Lamination embedded with hot-melt yarns provides a versatile approach for combining nanofiber membranes with conventional fabrics. It can be used to develop nanofiber-functionalized textiles for a wide range of applications, including fire protection, electrical insulation, sound absorption, filtration, marine applications, and more.

## 1. Introduction

Electrospun nanofiber membranes have large specific surface area, high porosity, small pore size, and high mechanical flexibility. They are controllable in terms of structure and are easy to functionalize. These unique properties make electrospun nanofibers have a wide range of applications, especially in the field of personal protection [1,2,3]. Research on nanofibers for personal protection has mainly focused on aerosol filtration, antibacterial applications, neutron/electromagnetic shielding, and thermal protection, which have wide applications in medical care, fire and flame retardants, and other fields [4,5,6].

Many researchers have reported improvements in personal protection using nanofiber membranes. For example, Liang et al. [7] fabricated a nanofiber membrane with impact resistance and radiation cooling functions by using poly(vinylidene fluoride-co-hexafluoropropylene), ionic liquid, and poly-borosiloxane (PBS) to form a core–shell nanofiber structure. The nanofiber membrane can attenuate more than 60% of the impact force, sense pressure stimuli with a sensitivity of up to 201.5 kPa^−1^, achieve a temperature sensing resolution of 0.1 °C, and reduce skin temperature by ≈17 °C under a solar intensity of 1 kW m^−2^. Zhang et al. [8] reported a composite nanofiber membrane prepared by electrospinning and vacuum filtration using MXene as the nanofiber core and Gd(OH)_3_/TPU as the sheath. The nanofiber membrane exhibited a high tensile strength of 22.94 MPa and excellent neutron (μ = 22.95 cm^−1^) and EMI (SE = 35.1 dB) shielding capabilities. Liu et al. [9] prepared a porous polyacrylonitrile (PAN)/polyvinylpyrrolidone (PVP) nanofiber membrane by electrospinning. The post-treatment to obtain a porous structure increased the specific surface area by 133.63% and the porosity by 391.02%, and a filtration efficiency of 98.76% and a pressure drop of 165 Pa were achieved.

However, nanofiber membranes typically suffer from low strength, making them unsuitable for stand-alone use [10,11]. As a result, nanofiber membranes are often used with a fibrous substrate to improve their application performance [12,13]. The combination with woven fabric takes full advantage of both materials, allowing for high air and moisture permeability to be maintained and high strength and durability to be achieved [14,15,16]. Many researchers have reported directly depositing freshly electrospun nanofibers onto a fabric substrate for personal protection applications. For example, Wang et al. [17] deposited polyvinylidene fluoride/polystyrene nanofibers onto silver-/zinc-coated cotton fabric for antibacterial purposes. The filtration efficiency for small aerosol dust (specific size of 0.3 μm, PM_0.3_) at 85 L/min was 99.1% at a pressure drop of 79.2 Pa. The retention rates within 20 min of contact for Escherichia coli and Staphylococcus aureus were 99.64% and 98.75%, respectively. Khajeh-Amiri et al. [18] prepared an antibacterial cotton fabric by depositing Ag_3_PO_4_-containing chitosan nanofibers directly onto a cotton fabric. The adhesion reduction rates of Staphylococcus aureus and Escherichia coli were 100% and 99.8%, respectively. However, direct deposition lacks a chemical bond between the nanofiber and the fabric substrate. Although they can be used to develop disposable protective garments, the poor bond and ease of delamination prevent their use in high-motion and multi-cycle applications.

Several investigators have reported improvements in nanofiber–substrate adhesion using various methods. Varesano et al. [19] improved the adhesion of nanofiber membranes to cotton and nylon fabrics by pretreating the fabrics with chemicals. After the treatment, the fabrics showed increased adhesion energy (0.93 and 0.86 J m^−2^ for cotton and nylon fabrics, respectively) compared to untreated fabrics (cotton: 0.58 J m^−2^, nylon: 0.57 J m^−2^). Zhao et al. [20] showed that plasma treatment of the fabric substrate before nanofiber deposition improved the stripping strength and stripping energy. After treatment with 200 W oxygen plasma for 3 min, the stripping strength of polyvinyl alcohol (PVA)/polyamide taffeta was increased by 3.21 cN and the stripping energy by 2.27 J m^−2^. However, increasing the fabric surface roughness had little effect on nanofiber adhesion. Despite these works, improving the interlayer adhesion of nanofibers with fabric substrates is still challenging.

Lamination is a widely used technique that has the advantage of being highly flexible in design. During lamination, the adhesive and fabric are bonded together by pressing at an elevated temperature [21]. Some nanofiber membranes have been bonded to conventional fabrics either as an adhesive or functional layer. Liu et al. [22] used a TPU/polyetherimide (PEI) nanofiber membrane as an adhesive to laminate two fabrics. The sandwich laminate showed a hydrostatic pressure of 38 kPa, high resistance to synthetic blood penetration, a high filtration efficiency of 99.7%, and an excellent water vapor transmission rate of 5.2 kg m^−2^ d^−1^. Li et al. [23] laminated a PU/SiO_2_ nanofiber membrane to polyester fabric. The prepared composite laminate had a hydrostatic pressure of 23.5 kPa and a water vapor transmission rate of 5.19 kg m^−2^ d^−1^. The lamination reduced the damage to the nanofibers during use. However, these laminations tend to reduce the permeability and flexibility of the fabric. It is important to develop an effective method that can not only improve the bond strength but also avoid a large reduction in permeability.

This study investigated two methods to laminate a nanofiber membrane with a woven nylon fabric: (1) a PAN nanofiber membrane and nylon woven fabric laminated using a TPU nonwoven adhesive, and (2) a PAN nanofiber/nylon fabric lamination with a yarn embedded by sewing. Lamination with the TPU nonwoven adhesive alone slightly improved the adhesion between the nanofiber membrane and the nylon fabric. However, it decreased the air permeability, with the degree of the decrease depending on the areal density of the TPU adhesive. As the areal density of the TPU increased from 10 g/m^2^ to 30 g/m^2^, the air permeability decreased from 107.6 mm/s to 43.4 mm/s. The lamination resulted in a slight increase in the filtration efficiency for oil aerosol particles (0.3 µm, PM0.3, at a flow rate of 32 L/min) to 96.4% with a pressure drop of 83 Pa. Embedding non-fusible yarns in the laminate increased the nanofiber/fabric adhesion and permeability, but the filtration efficiency and pressure drop were reduced to 74.4% and 38 Pa, respectively, due to numerous pinholes formed in the nanofiber layer during the sewing process. Conversely, incorporating fusible TPU yarns not only improved the interlayer adhesion by 175% compared to using TPU fabric adhesive alone but also increased the air permeability to 136.1 mm/s. However, the filtration performance (87.7%, 72 Pa) was slightly lower than that of the unlaminated nanofiber/fabric pack and the nanofiber/fabric pack because the TPU yarns sealed the pinholes during lamination. The method of laminating with embedded fusible yarns represents a potentially innovative approach to developing nanofiber/fabric laminates for a variety of textile applications.

## 2. Materials and Methods

### 2.1. Materials

Polyacrylonitrile (PAN, Mw = 275,000) was purchased from Spectrum Chemical Co., Ltd. (Shanghai, China). Nylon fabric (100–500-mesh) was purchased from Xiaosa Wire Mesh Co., Ltd. (Anping, China). Polyester (40 S/2), cotton (40 S/2), and nylon (150 D/3) yarns were purchased from Yi Tu (Zhejiang, China). TPU adhesive yarn (150 D) was purchased from Xingxia Polymer Products Co., Ltd. (Shanghai, China). TPU adhesive web (HWU98, 10–30 g/m^2^) was purchased from Xingxia Polymer Products Co., Ltd. (Shanghai, China). N, N-dimethylformamide (DMF) was purchased from Kemiou Chemical Reagent Co., Ltd. (Tianjin, China). All materials were used as received.

### 2.2. Electrospinning

A 12% (wt%) PAN solution was prepared by dissolving PAN in DMF at 60 °C. The PAN solutions were electrospun using a custom-built electrospinning apparatus with a roller collector to collect the nanofibers. During electrospinning, the applied voltage of the PAN solution, flow rate, spinning distance, and rotation speed of the roller collector were set to 18 kV, 0.5 mL/h, 12 cm, and 300 rpm. Electrospinning was performed under ambient conditions of 25 ± 5 °C and 20 ± 5% relative humidity. The nanofiber membrane sample was prepared by electrospinning for 1 h.

### 2.3. Laminations

#### 2.3.1. Hot-Press Lamination

A piece of PAN nanofiber membrane (thickness: 0.48 μm) was placed between two layers of nylon fabric, with a TPU adhesive web of varying areal densities (10–30 g/m^2^) inserted between the nanofiber and nylon layers. The multilayer fabric was hot-pressed using a laminator. The lamination temperature was controlled at 120 °C.

#### 2.3.2. Lamination with Infusible Yarn Embedded

The nanofiber membrane and nylon fabric were sutured (needle type: 14) with a yarn (polyester, cotton, and nylon). The distance between the stitch lines was controlled at 1 cm, 2 cm, 3 cm, 4 cm, and 5 cm. The stitched nanofiber/nylon fabric was then laminated to another piece of nylon fabric using a TPU adhesive tape as a binder. The lamination was performed at 120 °C for 1 s.

#### 2.3.3. Lamination with TPU Fusible Yarn Embedded

The procedure was similar to that outlined in Section 2.3.2, except that a TPU adhesive yarn was used to suture the nanofiber membrane and nylon fabric (needle type: 9).

### 2.4. Characterizations

The morphology was observed under a scanning electron microscope (SEM, Crossbeam 550, Carl Zeiss, Germany). The air permeability was tested by an automatic breathability tester (YG461H, Ningbo textile instrument factory, Ningbo, China) and referred to the standard “GB/T5453” [24]. The peel strength of the composite fabrics was tested using a universal tensile tester (Instron5969, Instron, Norwood, MA, USA) according to the standard “FZ/T 6011-2016” [25]. Air filtration performance was evaluated using an automated filter tester (AFC131, TOPAS, Dresden, Germany) with DEHS aerosol in the range of 0.2 µm to 4.59 µm. The diameter of the filters was 150 mm (test area, 176.7 cm^2^). During testing, the air velocity was set at 2.5 cm/s. Each test was repeated three times, and the filtration efficiency (η) and pressure drop (∆P) were the average of the tests.

## 3. Results

### 3.1. Preparation and Morphological Characterization of PAN Nanofiber Membranes

**Figure 1:**
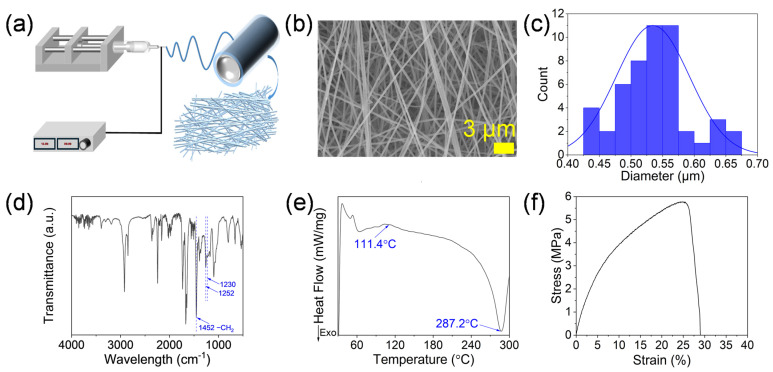
(**a**) Schematic diagram of the electrospinning process. (**b**) SEM images and (**c**) diameter distribution of the PAN nanofibers. (**d**) FTIR, (**e**) DSC, and (**f**) stress–strain curves of PAN nanofiber membrane.

Figure 1a schematically illustrates the preparation of the PAN nanofiber membrane. An SEM image of the PAN nanofiber membrane is shown in Figure 1b. The diameter distribution of the nanofiber membrane was measured by the software (Figure 1c), and the average diameter of the nanofiber was about 0.534 μm.

Figure 1d shows the FTIR spectra of the fabric. The PAN vibration bands at 1252 cm^−1^ and 1230 cm^−1^, corresponded to the PAN zigzag and 31-helical conformations, and the band at 1452 cm^−1^ was assigned to the CH_2_ bending vibrations in the PAN chain [26,27].

Figure 1e shows the DSC curves of the PAN nanofiber membrane in the N_2_ atmosphere. The glass transition of PAN was observed at about 110 °C, and the subsequent exothermic peak at 287.2 °C was due to the cyclization of the nitrile groups [26,28]. Figure 1f shows the PAN nanofiber membrane’s tensile properties. The membrane had a tensile strain of 5.78 MPa and a fracture strain of 24.4%.

**Figure 2:**
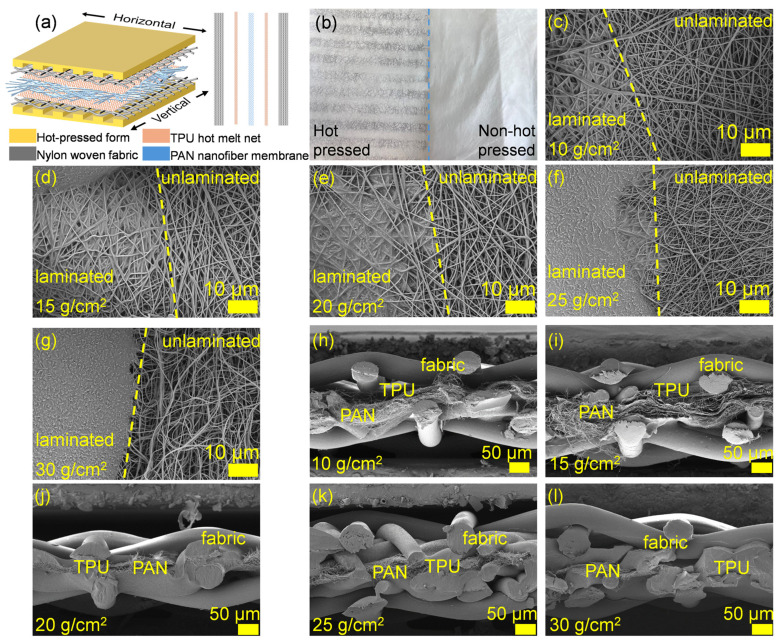
(**a**) Schematic illustration of the lamination methods, (**b**) photo of the hot-pressing grids. SEM images of the TPU adhesive with different areal densities, (**c**) 10 g/m^2^, (**d**) 15 g/m^2^, (**e**) 20 g/m^2^, (**f**) 25 g/m^2^, and (**g**) 30 g/m^2^ in the boundary zone of hot-pressed/non-hot-pressed and laminate cross-section, (**h**–**l**) cross-sectional SEM images of the PAN nanofiber/TPU adhesive/nylon laminates with the TPU areal densities of (**h**) 10 g/m^2^, (**i**) 15 g/m^2^, (**j**) 20 g/m^2^, (**k)** 25 g/m^2^, and (**l**) 30 g/m^2^.

Two main methods were used to laminate the PAN nanofiber with nylon fabric: (1) conventional lamination using a TPU adhesive tape as a binder, and (2) modified lamination using a TPU adhesive tape and a suture yarn for reinforcement. Figure 2a schematically illustrates the lamination process. In method 1, the lamination was selectively applied using a parallel grid hot-pressing method. As shown in Figure 2b, each grid was 6 mm wide.

### 3.2. Lamination of Nanofiber Membrane with Nylon Fabric Using Method 1

First, we focused on method 1 using the grids with different grid spacings (2, 4, 6, 8, 10 mm), nylon fabric mesh number (100-, 200-, 300-, 400-, 500-mesh), and TPU adhesive sheets with different areal densities (10, 15, 20, 25, 30 g/m^2^). When the 10 g/m^2^ TPU was used, only a very small amount of TPU adhered to the nanofiber surface in the hot-pressed zone. As a result, the hot-pressed and non-hot-pressed zones looked very similar (Figure 2c–g). As the increase in the TPU areal density (i.e., the thickness) increased, the wrapping phenomenon became obvious, and the pores within the nanofiber membrane were filled with TPU. When the areal density reached 30 g/m^2^, the TPU formed a polymer film on the surface of the nanofiber membrane. This made the hot-pressed area transparent. As the areal density of TPU continued to increase, the hot-pressed zone tended to form a thicker film that was harder and less flexible. From the cross-section SEM image of the laminate, it was found that when the surface density of TPU was 10–15 g/m^2^, the melted TPU was distributed in the contact part of the yarn and the nanofiber (Figure 2h,i). Both the nanofibers and nylon yarns remained in their original state. As the TPU surface density increased to 20 g/m^2^, the nanofibers and yarns were tightly bonded by the TPU and no longer remained fluffy (Figure 2j). When the TPU surface density continued to increase, the pores between the nanofibers and the yarn were further reduced (Figure 2k,l).

#### 3.2.1. Air Permeability

**Figure 3:**
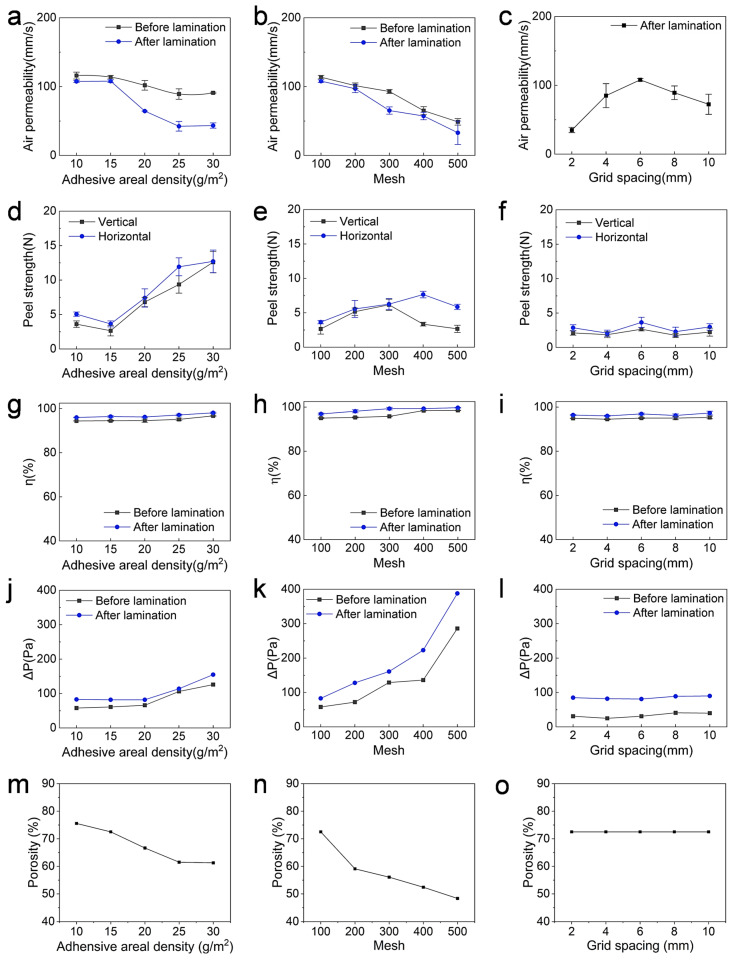
(**a**–**c**) Air permeability, (**d**–**f**) peeling strength, (**g**–**i**) PM0.3 η, (**j**–**l**) ∆P, and (**m**–**o**) porosity of the laminated fabrics with different TPU adhesive areal density, mesh, and grid spacing. Mesh = 100, grid spacing = 6 mm for (**a**,**d**,**g**,**j**,**m**); TPU areal density = 15 g/m^2^, grid spacing = 6 mm for (**b**,**e**,**h**,**k**,**n**); TPU areal density = 15 g/m^2^, mesh = 100 for (**c**,**f**,**i**,**l**,**o**). Flow rate: 32 L/min for g^−l^.

Figure 3a shows the effect of the TPU areal density on the air permeability of the laminated fabrics. Before lamination, there was only a slight change in air permeability (from 116.04 mm/s to 91.02 mm/s). After lamination, the air permeability decreased. As the TPU areal density increased from 10 to 30 g/m^2^, the air permeability decreased from 107.6 mm/s to 43.4 mm/s. This can be explained by the blocking effect of TPU on the micropores within the nanofiber membrane and nylon fabric.

The previous results were based on the 100-mesh nylon fabric. We also investigated the effect of the pore size of the nylon substrate on the air permeability. Without lamination, the air permeability of the nanofiber membrane/TPU adhesive web/nylon fabric decreased from 113.7 mm/s to 48.9 mm/s as the mesh size of the fabric pore aperture increased (Figure 3b). This is reasonable because a larger aperture and smaller mesh size mean that larger pores provide less airflow resistance and therefore greater air permeability. After hot pressing, the air permeability was reduced, but the overall trend was similar to that without hot pressing. The decrease was from 107.7 mm/s to 33.1 mm/s.

Figure 3c shows the air permeability of the hot-pressed laminated fabric at different mesh spacings. As the pitch increased from 2 mm to 6 mm, the air permeability increased from 35 mm/s to 108 mm/s. The air permeability decreased as the mesh spacing was further increased. When the spacing was increased to 10 cm, the permeability was reduced to 72 mm/s.

#### 3.2.2. Peel Strength

Figure 3d shows the peel strength of the laminated fabrics with TPU at different areal densities. Both the horizontal and vertical peel strengths were tested. The peel strength increased with the increase in the TPU areal density in both peel directions. The horizontal peel strength changed from 3.6 N to 12.6 N, while the vertical peel strength increased from 5.1 N to 12.7 N.

The peel strength was also affected by the mesh size of the nylon fabric (Figure 3e). As the mesh size of the nylon woven fabric increased from 100 mesh to 300 mesh, the horizontal peel strength increased from 2.6 N to 6.3 N, while the vertical peel strength increased from 3.6 N to 6.2 N. At 400 mesh, the laminated fabric achieved a vertical peel strength of 3.3 N and a horizontal peel strength of 7.7 N.

Figure 3f shows the peel strength of the laminated fabric using the hot-pressed fabric with different mesh spacing. With the increase in the mesh spacing, the peel strength showed no obvious change, with the peel strength being about 2.2 N in the horizontal direction and 2.1 N in the vertical direction.

The above results showed that the peel strength in the horizontal direction was higher than that in the vertical direction. This is because the bond along the horizontal direction had a larger bond cross-section than that in the vertical direction.

#### 3.2.3. Oil Mist Filtration Properties

In this study, we measured the filtration properties against an oil aerosol to understand the personal protection ability of the laminated fabrics. As shown in Figure 3g,j, the filtration efficiency and pressure drop of the laminated fabrics did not change significantly as the areal density of the TPU adhesive increased. Without lamination, the fabric pack achieved a η of 95% at a ∆P of 80 Pa. After lamination, both the filtration efficiency and pressure drop increased significantly. As the areal density of the TPU increased from 10 g/m^2^ to 30 g/m^2^, the η increased from 95.5% to 98.1% and the ∆P increased from 83 Pa to 155 Pa.

The filtration properties of the laminated fabrics were investigated using the nylon substrate with different mesh sizes (Figure 3h,k). Using the TPU adhesive with an areal density of 15 g/m^2^ and hot pressing with 6 mm mesh size, the η increased from 96.9% to 99.7% and the ∆P increased from 118 Pa to 388 Pa as the mesh size increased.

Figure 3i,l show the filtration performance of the nanofiber/nylon fabric laminated with different mesh sizes. As the mesh size increased from 2 mm to 10 mm, there was no obvious change in the filtration performance. The laminated fabric maintained a η value at about 97%, and the ∆P was about 85 Pa.

#### 3.2.4. Porosity

The porosity of the PAN nanofiber membrane and nylon fabric (100-mesh) was 86.8% and 82.17% (for 100-mesh fabric). The porosity of the nanofiber–nylon fabric laminates decreased with the increase in the areal density (i.e., thickness) of TPU. As the areal density of TPU increased from 10 g/m^2^ to 30 g/m^2^, the porosity of the laminates decreased from 75.56% to 61.26%.

The porosity was also affected by the mesh size of the nylon fabric. As the mesh size of the nylon fabric increased from 100 mesh to 500 mesh (porosity 37.58%), the porosity of the laminate decreased from 72.51% to 48.35%. However, the structure of the hot-pressing plate had little effect on the porosity of the laminate, and the porosity remained at 72.51% with the variation in the mesh size of the hot-pressing plate.

These effects could be due to the infusion of TPU adhesives into the nanofiber and nylon fabric structures, which filled the local pores, resulting in a reduction in the porosity. A thicker TPU layer would result in more pores being filled, thus reducing the porosity. The denser nylon fabric, which had a large mesh and smaller pores, could exert more pressure on the TPU, increasing its infusion into the porous structures.

### 3.3. Lamination of Nanofiber Membrane with Nylon Fabric Using Method 2

Method 2 used a similar hot-pressing process except that one of the TPU nonwovens was replaced with a yarn for the nanofiber/nylon lamination. Prior to hot pressing, the yarn was used to sew the nylon fabric and nanofiber membrane together. The sewn nanofiber/fabric was then laminated to another piece of nylon fabric using a TPU sheet as an adhesive.

In Section 3.2.1, the lamination using the nonwoven adhesive with an area density of 15 g/m^2^, a nylon fabric mesh number of 100 mesh, a stencil grid spacing of 6 mm, and hot pressing for 1 s at 120 °C showed the best results. Therefore, these parameters were selected for the studies of method 2. Initially, nylon, readily available cotton, and polyester yarns were used as the embedding yarns. The basic parameters of the yarns are listed in Table 1.

#### 3.3.1. Incorporation of Infusible Yarns

**Figure 4:**
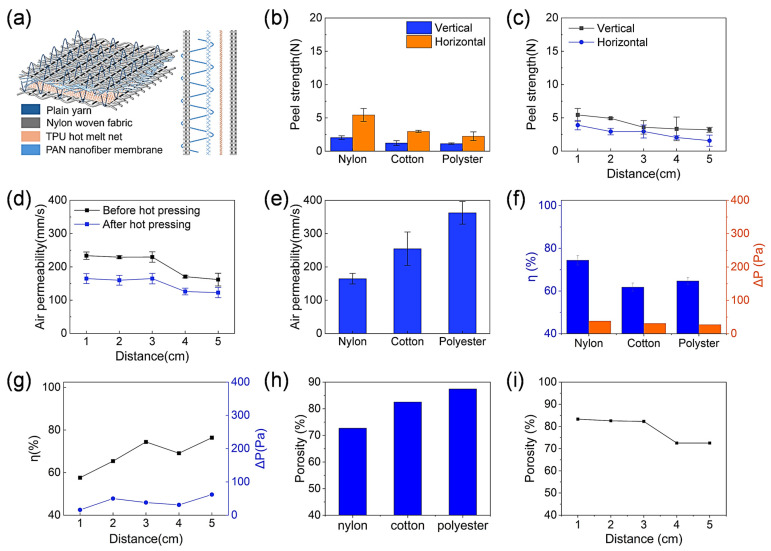
(**a**) The structure of the yarn-embedded nanofiber/fabric laminate. (**b**) Peel strength, (**e**) air permeability, (**f**) η and ∆P, (**h**) porosity of laminates with nylon, cotton, and polyester yarns embedded. (**c**) Effect of embedding distance on peel strength, (**d**) air permeability, (**g**) η and ∆P, and (**i**) porosity of laminate fabric. Flow rate: 32 L/min for (**f**–**i**).

Figure 4a illustrates the structure of the yarn-embedded nanofiber/fabric lamination. Three different yarns were embedded separately. Figure 4b shows the peel strength of the laminated fabrics. Vertically, the fabric based on nylon yarns had the highest peel strength, about 2.1 N. The laminated fabrics embedded with cotton and polyester yarns had a peel strength of 1.2 N and 1.1 N, respectively. The lower peel strength associated with using cotton and polyester yarns was presumably attributed to the larger diameter of the nylon yarns. During hot pressing, more TPU was bonded to the nylon yarn, increasing the bonding area between the yarn and the nylon fabric, thereby improving the peel strength. Similarly, in the horizontal direction, the nylon fabrics had the highest peel strength at approximately 5.4 N, which was much higher than that of the fabrics embedded with cotton and polyester yarns, which were 2.9 N and 2.2 N, respectively.

The effect of the embedment distance on the peel strength was investigated (Figure 4c). As the embedment distance increased, the horizontal peel strength decreased from 3.9 N to 1.6 N. This could be due to the decrease in the number of embedded wires in the same area as the distance from the embedded wires increased. As a result, the dispersing force of the yarn during the stretching process was reduced, resulting in a decrease in the peel strength. However, the horizontal peel strength of the composite fabric decreased from 5.4 N to 3.2 N.

Figure 4d shows the air permeability of the laminated fabrics (nylon yarn). Regardless of the lamination, the nanofiber/fabric packages increased the air permeability when the yarns were embedded. The air permeability decreased as the embedding distance increased. This is because the number of microholes formed by the embedded wires decreased as the distance from the embedded wires increased. When the embedding distance was 1 cm, the air permeability before and after hot pressing was 233.6 mm/s and 164.8 mm/s, respectively. When the embedment distance increased to 5 cm, the air permeability decreased to 161.8 mm/s and 122.6 mm/s before and after hot pressing, respectively. Figure 4e shows that the air permeability of the three fabrics was 164.8 mm/s, 254.8 mm/s, and 362.8 mm/s for the polyester, cotton, and nylon yarns, respectively. The laminated fabric embedded with nylon yarn showed a lower air permeability than the others due to the larger yarn diameter.

Figure 4f compares the filtration performance of the three embedded fabrics. The laminated fabric embedded with nylon yarns had the highest η and ∆P of 74.4% and 38 Pa, respectively. The cotton and polyester fabrics had similar η and ∆P values of approximately 61.8% and 64.7% and 31 Pa and 27 Pa, respectively. The higher filtration efficiency can also be attributed to the fact that the coarse yarns filled the microholes, thus reducing the actual hole size.

Figure 4f compares the porosity of the three laminates embedded with different yarns. The one embedded with polyester yarn had the highest porosity, reaching 86.99%. The porosity of those embedded with nylon and cotton yarns was 82.3% and 82.66%, respectively. The higher porosity was attributed to the insufficient filling of the pores by the fine yarn, increasing the pore size. Figure 4g examines the effect of the buried wire distance on the porosity of the laminated fabric. When the embedment distance was 1 cm, the porosity was 83.3%, and as the embedment distance increased, the laminate porosity decreased to 72.51%. This was because an increase in the embedding distance led to a reduction in the number of pinholes, thereby reducing the aperture of the fabric.

#### 3.3.2. Incorporation of Fusible TPU Yarns

**Figure 5:**
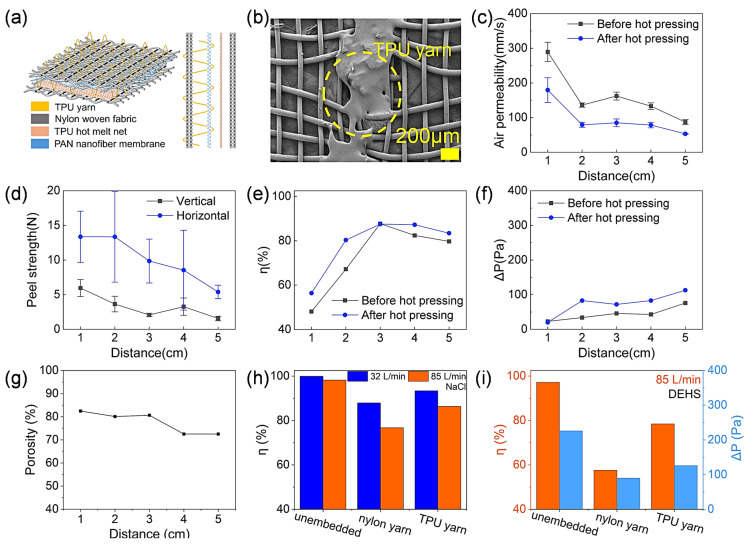
(**a**) Structure diagram of embedded TPU yarn composite fabric. (**b**) SEM topography of pinhole after hot pressing. (**c**) Air permeability, (**d**) stripping strength, (**e**) η, (**f**) ∆P, and (**g**) porosity of the composite fabric at different embedding spacings. Filtration efficiency and pressure drop of (**h**) unembedded yarn, nylon yarn, and TPU yarn laminates for 0.3 μm NaCl and (**i**) DEHS aerosol at flow rates of 32 L/min and 85 L/min, respectively. TPU areal density = 15 g/m^2^, grid spacing = 6 mm, mesh = 100, burial distance = 3 cm for (**h**,**i**).

Figure 4g shows the effect of the burial distance on the filtration performance. As the burial distance increased, the η increased from 57.6% to 76.4%, while the ∆P increased from 16 Pa to 62 Pa. This can also be attributed to the increase in the number of micropores caused by the embedding of the filaments. TPU yarn was embedded in the laminated fabrics in a similar way to the PET, cotton, and nylon yarns. Unlike these infusible yarns, the TPU could be melted during hot pressing to allow the melt to fill the pinholes that are formed by the sewing needle. In addition, the melted TPU yarns could be bonded between the nylon fabric and the PAN nanofiber, improving the interlayer bonding. Figure 5a schematically shows the lamination structure with the TPU yarn. After hot pressing, the TPU yarn melted and blocked the pinholes (Figure 5b).

The composite fabric embedded with TPU yarns showed a decrease in air permeability with the increase in the buried yarn spacing (Figure 5c), the trend of which was consistent with that of the embedded conventional yarns. Hot pressing resulted in a significant decrease in air permeability after hot pressing. As the embedding distance increased from 1 cm to 5 cm, the air permeability decreased from 179.5 mm/s to 53.3 mm/s, which can be attributed to the reduction in micropores.

Figure 5d shows the effect of the burial distance of the TPU yarn on the peel strength of the laminated fabric. With the increase in the burial distance, the vertical peel strength decreased from 5.9 N to 1.7 N. However, the peel strength in the horizontal direction did not change much and remained at about 9.8 N. Compared with ordinary yarns, the peel strength of the composite fabrics embedded with TPU yarns was significantly improved, which was attributed to the fact that the fused TPU yarns increased the bonding area between the nanofiber membranes and nylon fabrics.

Figure 5e,f show the effect of the burial distance on the filtration characteristics. As the burial distance increased, the η increased from 56.4% to 87.5% and the ∆P increased from 20 Pa to 113 Pa.

The effect of the burial distance on the porosity of the laminated fabric is shown in Figure 5g. As the burial distance increased from 1 cm to 5 cm, the porosity of the fabric decreased from 82.47% to 72.5%.

The filtration performance of the laminate was tested on salt particles. Compared to oil particles, solid particle aerosols tend to carry a static charge and have a stronger attraction to nanofibers. At a flow rate of 32 L/min, the filtration efficiency of the unembedded yarn, nylon yarn, and TPU yarn laminates for 0.3 μm NaCl reached 99.94%, 87.92%, and 93.36%, respectively (Figure 5h). At a flow rate of 85 L/min, the filtration efficiency of the unembedded yarn, nylon yarn, and TPU yarn laminates decreased to 98.19%, 76.74%, and 86.36%, respectively. At this air flow rate, the filtration efficiencies for 0.3 μm oil particles were 97.15%, 57.6%, and 78.45% (Figure 5i), and the pressure drops were 226 Pa, 90 Pa, and 126 Pa, respectively.

## 4. Discussion

**Figure 6:**
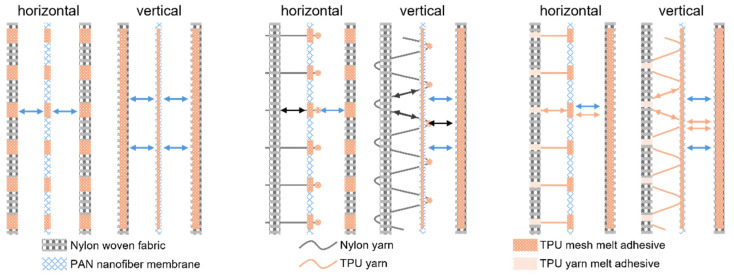
The effect of the embedded yarn on the peel strength of the laminated fabric.

Typically, the modest mechanical properties of nanofiber membranes pose a challenge when integrating them with conventional textiles. Our research has shown that by applying a fabric adhesive during lamination, the adhesion between nanofibers and conventional fabrics can be significantly improved. In addition, incorporating yarn into the laminated fabric and using a fabric adhesive to bond the yarn to both the fabric and the nanofibers significantly increases the bond strength. Figure 6 shows the effect of yarn incorporation on the peel strength of the laminated fabric.

Compared to using only fabric adhesive in the laminate, the addition of yarn effectively distributes the force on the nanofiber membrane, thereby increasing the overall stripping strength of the laminate. In addition, compared to nylon yarns, fusible yarns allow for more force to be reduced on the nanofiber membrane during stripping because the melting of the yarns increases the area of adhesion between the nanofiber and the fabric. This allows for the stripping strength of the laminate to be the highest.

Our approach is innovative in its use of embedded yarns to improve adhesion between nanofiber membranes and fabrics and fusible yarns to improve air permeability without compromising the high air filtration efficiency. This innovation expands the potential applications of nanofiber membranes in textile products. The improved air permeability and bond strength make the laminated fabric more wearable and durable, while the improved filtration performance suggests a higher level of protection against particulate contamination.

However, our method is not without its drawbacks, most notably the problem of pinholes in the nanofiber layer due to the use of a sewing method for yarn incorporation. Although this adverse effect can be largely offset by using fusible TPU yarns that seal the pinholes during the hot-pressing process, exploring alternative methods to stitching for yarn incorporation could prevent pinhole formation, which is worthy of further research.

## 5. Conclusions

We developed a novel method to laminate nanofiber membranes with a common fabric by incorporating yarn. The incorporation of fusible yarn effectively improved the interlayer adhesion of the nanofiber membranes and fabric. As a result, the laminate had an air permeability of 136.1 mm/s, a peel strength of 9.9 N, and a filtration efficiency of 87.7% for PM0.3 with a pressure drop of 72 Pa at a flow rate of 32 L/min. This could form a novel approach to developing nanofiber/fabric laminates for a variety of textile applications.

## Figures and Tables

**Table 1:** Basic properties of yarn.

Yarn	Count	Size (μm)	Strength (N)
Nylon	150D/3	180	35.6
Cotton	40S/2	120	11.8
Polyester	40S/2	50	12.0

## Data Availability

Data are available upon request from the respective authors.
